# Instruments evaluating the self-directed learning abilities among nursing students and nurses: a systematic review of psychometric properties

**DOI:** 10.1186/s12909-017-1072-3

**Published:** 2017-11-25

**Authors:** Lucia Cadorin, Valentina Bressan, Alvisa Palese

**Affiliations:** 1 CRO Aviano National Cancer Institute, Via F. Gallini, 2, 33081 Aviano, Pordenone Italy; 2 University Hospital of Udine, Via Pozzuolo, 330, 33100 Udine, Italy; 3 Department of Medical and Biological Sciences, University of Udine, Viale Ungheria, 20, 33100 Udine, Italy

**Keywords:** Self-directed learning, Systematic psychometric review, Nursing education, Nursing student, Nurse, Tool, Questionnaire, Assessment

## Abstract

**Background:**

Modern healthcare institutions are continuously changing, and Self-Directed Learning (SDL) abilities are considered a prerequisite for both nursing students and nurses in order to be proactive about these demanding challenges. To date, no systematic reviews of existing instruments aimed at detecting and critically evaluating SDL abilities have been published. Therefore, the aims of this review are: 1) identify the instruments for assessment of SDL abilities among nursing students and nurses; 2) critically evaluate the methodological studies quality; and 3) compare the psychometric properties of the available instruments.

**Methods:**

A psychometric-systematic-review was performed. CDSR, CINAHL, ERIC, MEDLINE, PROSPERO, SCOPUS databases were searched without restrictions in time and setting. All primary studies involving nursing students or nurses, written in English and aimed at validating SDL assessment tools, were included. Studies retrieved were evaluated according to the COnsensus-based-Standards for the selection of health Measurement-INstruments (COSMIN) panel. Study inclusion, data extraction and quality assessment were performed by researchers independently.

**Results:**

Eleven studies were included and four tools based on Knowles’s theory have emerged: 1) the Self-Directed Learning Readiness Scale; 2) the Self-Directed Learning Readiness Scale for Nursing Education; 3) the Self-Rating Scale of Self-Directed Learning, and 4) the Self-Directed Learning Instrument. A few psychometric properties have been considered in each study, from two to four out of the ten required. The quality of the methodologies used was in general, from fair to poor with the exception of one instrument (the Self-Directed-Learning-Instrument). The psychometric proprieties that emerged across the tools were good in general: the Cronbach α was from 0.73 to 0.91; structural validities have also reported good indexes both in the explorative and in the confirmative factor analyses.

**Conclusions:**

On the basis of the findings, the Self-Directed-Learning-Instrument can be recommended for the assessment of SDL abilities among nursing students and nurses, given the excellent methodology quality adopted in estimating the psychometric properties. However, rigorous study designs aimed at estimating psychometric properties of tools in wide samples of nursing students and nurses across different stages of professional life, from undergraduate education to professional maturity, in different cultural, educational, and work settings, are strongly recommended.

## Background

Modern healthcare institutions are continuously in motion under the stimuli of different challenges, where emerging new health problems, the need to implement new knowledge developed thanks to research and the required cost-effective interventions are only some examples [1]. All health-care professionals are required to be proactive in these changes [2, 3]. How to prepare and maintain a future workforce capable of dealing with these rapid changes, using new knowledge [4, 5] thus functioning as self-directed learners, has been highlighted in the agenda of both higher educational institutions (HEIs) [6] and continuing educational strategies [7]. Specifically, in the nursing discipline, the increased evidence available as well as the complexity of patients’ problems, in adjunction to the limited time devoted to education lasting from three to four years around the world, have increased the need to evaluate Self-Directed Learning (SDL). Finding reliable and valid instruments capable of detecting baseline levels and subsequent improvements among nursing students or nurses exposed to different teaching strategies, as well as to compare effectiveness across different strategies in undergraduate, advanced and continuing education agencies, has been established as a priority in the nursing discipline [6, 7].

Some tools have been developed to evaluate Self-directed learning (SDL) abilities which have been considered a prerequisite for both students and health-care professionals [5, 8]. Dewey [9] was the first scientist who defined the mission of educational agencies with regard to the SDL as ‘*the individual’s growth*’ suggesting that ‘*the educator should be the one who guides, but does not control the process of learning*’. Later, Knowles [10] recognized as the father of the andragogical theory as the art and the science of adult learning, has defined the SDL abilities or readiness as:

‘*A process in which individuals take the initiative, with or without the help of others, in diagnosing their learning needs, formulating learning goals, identifying human and material resources for learning, choosing and implementing appropriate learning strategies, and evaluating learning outcomes*’ [10].

In the nursing discipline, studies attempting to evaluate the SDL abilities were performed initially by Crook and Dixon [11, 12]. From these early studies, researchers’ interest has increased, aiming at identifying SDL abilities and their development over time [4, 13] also as a consequence of tailored education strategies adopted [14, 15].

However, despite the increased attention, only three reviews have been conducted so far [5, 16, 17]. By including studies from 1975 to 2002 O’Shea [17] explored the definitions of SDL available by conceptualizing the nature of SDL, the implicated learning styles and the ability to be self-directed in learning. According to the findings, a student-centered approach may facilitate an increase in SDL abilities [17] while no suggestions have been made with regard to the tools measuring the SDL abilities.

Murad and Varkey [16], in their overview, which included studies from 1980 to 2005, identified the key principles of SDL: the educator as a facilitator, the identification of learning needs, the development of learning objectives, the commitment established with a learning contract, the identification of resources, the implementation process, and the learning evaluation. In accordance with these findings, several educational programmes applying these principles have been established to date, but little evidence has been discovered on the effectiveness of these key principles on SDL abilities [16]. However, also in this study, no suggestions with regard to the tools measuring SDL abilities, have been recommended.

More recently, Murad and colleagues (2010), in their systematic review including studies from 1975 to 2009, analyzed the effectiveness of SDL on knowledge, skills and attitude improvements. Moderate-quality evidence was retrieved; in accordance to their findings, SDL is associated with moderate improvement in knowledge as compared with traditional methods of teaching [5]. For this reason, authors have recommended educators to: involve learners in choosing learning resources and strategies; consider SDL as an effective strategy for more advanced learners (e.g. in the last years of nursing or medical school rather than in basic school); and to consider SDL when the learning outcome falls in the knowledge domain [5].

On the basis of the above-mentioned studies, to date no systematic review on tools aiming at detecting and critically evaluating SDL abilities have been found in literature. Therefore, with the intent to cover the gap in the available literature, the aims of this review are: 1) to identify instruments evaluating self-directed learning abilities among nursing students and nurses that have undergone validation processes; 2) to evaluate critically the quality of the methods used in ascertaining psychometric properties; and 3) to compare the estimated psychometric properties of the instruments available.

## Methods

### Study design

A psychometric systematic reviekw was performed in 2016 on the basis of the following guidelines:

- the Cochrane Guidelines on Effective Practice and Organization of Care (EPOC) [18];
- the Preferred Reporting Items for Systematic reviews and Meta-Analyses (PRISMA) statement for systematic reviews [19];
- The COnsensus-based Standards for the selection of health status Measurement Instruments (COSMIN) checklist [20, 21].

The psychometric systematic review protocol was registered on the international prospective register of systematic reviews (PROSPERO, registration number: CRD42016039613).

### Data bases and search strategy

On a preliminary basis, the search question was defined in terms of population, interventions, comparison, outcomes and study designs (PICOS) [19], as reported in Table 1.

**Table 1:** Search questions

Population	Nursing student, nurse, nurs*
Intervention	Assessment, assess*, evaluation, tool, instrument
Comparison	None
Outcome	Measures of self-directed learning
Study	Psychometric or validation studies Randomized studies Non-randomized studies Prospective and retrospective studies Cohort studies Case-control studies Cross-sectional studies Before and after comparison studies Observational studies Surveys

Legend: *PICOS* Population, Intervention, Comparison, Outcome Study

*All words with root "nurs" or "assess"

Then, a systematic search of the literature was performed in the following databases: the Cochrane Database of Systematic Reviews (CDSR), the Cumulative Index of Nursing and Allied Health (CINAHL), the Education Resources Information Centre (ERIC), MEDLINE, PROSPERO Database (International prospective register of systematic reviews) and Scopus. Grey literature was also searched via Google, aiming at retrieving potential studies. No restriction with regards to time, setting and language was applied.

The following research terms were used: nursing student, nurse, nurs* professional, assessment, assess*, evaluation, tool, self-directed learning, combined as MESH and text words as reported in Table 2.

**Table 2:** Search strategy

Database	Search strategy
CDSR CINAHL	“self-directed learning” (“self-directed learning” AND (nurse OR nursing OR professional) AND (assessment OR evaluation OR tool OR instrument))
ERIC	((nursing students OR nurse professionals OR nurse OR nursing) AND (self-directed learning) AND (evaluation OR assessment OR tool OR instrument))
MEDLINE	(“self-directed learning” AND (assessment OR evaluation OR assess* OR tool OR instrument) AND (nurs* OR nurse OR nursing) AND (professional OR student))
PROSPERO	“self-directed learning”
SCOPUS	((nursing students OR nurse professionals OR nurse OR nursing) AND (self directed learning) AND (evaluation OR assessment OR tool OR instrument))

Legend: *CDSR* Cochrane Database of Systematic Reviews, *CINAHL* Cumulative Index to Nursing and Allied Health, *ERIC* Education Resources Information Centre, *MEDLINE* U.S. National Library of Medicine, *PROSPERO* International Prospective Register of Systematic Reviews – Centre for Review and Dissemination University of York *SCOPUS* Bibliographic database by Elsevier

For each included study, a) the reference list, and b) the descendant citations were checked using the Scopus database.

The search strategy was developed and performed in January 2016 (1st January 1970-31th January 2016) and repeated in May 2016 (1st January 2016 - 31th May 2016) by a researcher author (LC) and checked by a second researcher (VB). Some authors [13, 22] were also contacted via email aiming at exploring the availability of unpublished studies.

### Study selection

All primary studies involving nursing students and nurses, aimed at validating the SDL instruments, were eligible. Included were those 1) primary studies using quantitative methods, 2) those describing psychometric properties of tools’ measuring SDL abilities, or 3) those reporting SDL tool validity data in specific manuscript sections (e.g. data collection process) as randomized or non-randomized studies (before and after comparison, prospective and retrospective cohort, case-control, and cross-sectional), observational studies and surveys, and 4) published in English.

Thus, excluded were those studies 1) adopting qualitative methods, 2) not reporting data on psychometric properties of the SDL tool adopted, and 3) not accessible.

### Search outcomes

Researchers read carefully the titles and the abstracts of the eligible studies, analysing each of them against the inclusion and the exclusion criteria. When both the title and the abstract were unclear, researchers retrieved and read the full texts, as well as contacting authors, e.g. Kocaman and colleagues [13], who provided data requested. Those eligible studies published by one or more researchers performing this psychometric systematic review were read and checked against the inclusion criteria by a researcher not involved in the publication.

The process was conducted preliminarily in an independent fashion, then researchers agreed upon the study to be included; any disagreement was resolved through discussion among the researchers. The inter-rater agreement for the inclusion of the full texts was assessed and Kappa = 0.739 (*p* < 0.001) emerged showing a substantial strength of agreement between reviewers [23].

**Fig. 1:**
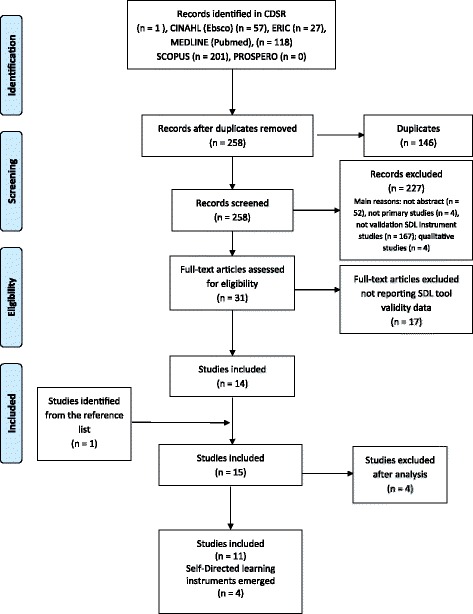
Flow Diagram according to Preferred Reporting Items for Systematic reviews and Meta-Analyses (PRISMA [19])

Databases provided 403 records; from this initial list, 145 were removed given that they were duplicates while 227 were excluded according to the inclusion and the exclusion criteria. Thus, a total of 31 studies remained eligible; 17 were subsequently excluded because they were not relevant to the research objectives while one study that emerged from the reference list was added. With regard to the remaining 15 studies, four were excluded [2, 24–26] given that instrument validation was not among the study aims. Consequently, a total of 11 studies were included as reported in Fig. 1.

### Quality appraisal

The methodological quality of the studies included was assessed by using the COSMIN checklist [20, 21]. The so-called ‘Step 2’ according to the COSMIN checklist was firstly considered to determine whether the statistical method used in the study was based on Classical Test Theory (CTT) or Item Response Theory (IRT). Then the methodological quality used by each study in estimating the psychometric properties (by 5–18 items) was evaluated: internal consistency, reliability, measurement error, content validity, structural validity, hypothesis testing, cross-cultural validity, criterion validity and responsiveness [21]. Each item included in the above-mentioned boxes is based on a four-point Likert scale (from poor to excellent) [27].

The evaluation was performed in couples, in two rounds, by reading and re-reading the studies included; in the first round, there was an independent evaluation, in the second by comparing the findings. Those studies performed by researchers (see authors) were evaluated by a third researcher as an external reviewer. Disagreements were discussed and resolved with discussion among researchers or by involving an external supervisor (statistician). Correlations of the scores attributed to each item and in each box of the COSMIN checklist by each couple of researchers, was assessed by calculating the Spearman’ coefficient (*r*_*s*_). Findings reported from strength to excellent agreement (box A: *r*_*s*_ = 0.902, *p* = < 0.001; box E: *r*_*s*_ = 0.819, *p* = 0.013).

### Data extraction and synthesis

Data was extracted using a predesigned and piloted template including the following variables: 1) identification of the study (author, title, citation), country/countries where the study was performed and the year(s); 2) theoretical model on which the tool was based; 3) SDL instrument features (e.g. items, dimensions, metrics [e.g. Likert scale from 1 to 5] and language); 4) characteristics of the study (aim, study design, sampling methods used and data collection process [e.g. self-administration of the tool] and when the data collection was performed); 5) participants and response rate; female proportion and age of participants; 6) psychometric properties of the instruments according to the COSMIN checklist [20, 21], and 7) summary of the COSMIN evaluation emerged from good to poor [20, 21].

## Results

### Validated instruments evaluating SDL abilities

A total of 11 studies published between 1985 and 2016 emerged, as reported in Table 3, reporting data collected from 1978 to 2014. Three studies were conducted in Italy [28–30], two in Australia [31, 32] and Taiwan [24, 33], one in Canada [20], China [34], Japan [3], and United Kingdom [35].

**Table 3:** Study characteristics (*n* = 11)

Tools	Author, country,	Aim(s)	Study design, sampling method, data collection year	Participants/response rate; female (%) and age	Reference Theory	Items (number), dimensions (number), metrics and score range; language
SDLRS	Crook, [11] Canada	1 – To investigate the predictive validity of the SRSSDL among first-year nursing students 2 – To test the correlation between SDLRS and alternative measures of self-directedness (nominations such as self-directed learners by peers and faculty, entry grades from high school, grades received at the end of the first-year in five subjects	Design: not reported Sampling: not reported Data collection: 1978–1979	63 nursing students enrolled in the nursing programme/90% Female: 93% Age: range 19–23 years	Knowles, 1975	Items: 57 Dimensions: not reported 5-point Likert scale Scores: from 57 to 285 Language: not reported
SDLRSNE	Fisher et al., [32] Australia	To develop and pilot an instrument measuring SDL readiness	Design: not reported Sampling: convenience Data collection year: not reported	201 nursing students/not reported Gender: not reported Age: not reported	Knowles, 1975	Items: 40 Dimensions: three (self-management, desire for learning, self-control) 5-point Likert scale Scores: from 40 to 200 Language: not reported
	Fisher and King, [31] Australia	To re-examine the factor structure of the SDLRSNE	Design: cross-sectional Sampling: not reported Data collection: not reported	227 first-year undergraduate nursing students/not reported Gender: not reported Age: not reported	Not reported	See above
	Fujino-Oyama et al., [3] Japan	To examine the reliability and validity of the Japanese version of the SDLRSNE when used among graduate nursing students	Design: cross-sectional Sampling: not reported Data collection: 2014	376 nursing students/24.2% Female: 88.3% Age: average 38.6 years	Knowles, 1975	See above
SRSSDL	Williamson, [35] United Kingdom	To develop and test the SRSSDL	Design: descriptive study Sampling: convenience Data collection year: not reported	30 nursing students/not reported Gender: not reported Age: range 20–25 years	Knowles, 1975	Items: 60 Dimensions: five (awareness, learning strategies, learning activities, evaluation, interpersonal skills) 5-point Likert scale Scores: from 60 to 300 Language: English
	Cadorin et al., [28] Italy	1 – To evaluate cross-cultural validity and test–retest reliability of SRSSDL 2 – To evaluate internal consistency of the SRSSDL	Design: not reported Sampling: two convenience samples Data collection: 2007	a.41 nurses/100% Age: average 43 years b. 334 nurses, health-care assistants, paediatric nurses, midwives/83.5% Female: 89.5% Age: average 41.2 years	Not reported	See above Language: Italian
	Cadorin et al., [29] Italy	To evaluate the factor structure of the Italian version SRSSDL	Design: cross-sectional Sampling: convenience Data collection year: 2009–2010	844: 182 nursing students, 453 RN; 141 RTs, 68 RTs students/ 67.5% Female: 75.2% Age: average 34.7 years	Knowles, 1975	See above
	Cadorin et al., [30] Italy	To establish the concurrent validity between SRSSDL and the SDLI used among undergraduate nursing students	Design: concurrent validity study Sampling: not reported Data collection: 2014	428 nursing students/90% Female: 78.5% Age: average 22 years	Knowles, 1975	Items: 40 Dimensions: eight (awareness, attitudes, motivation, learning strategies, methods, and activities, interpersonal skills, constructing knowledge) 5-point Likert scale Scores: from 40 to 200 Language: Italian
SDLI	Cheng et al., [24] Taiwan	1 – To develop an instrument to measure the SDL abilities of nursing students 2 – To test the validity and reliability of the SDLI	Design: not reported Sampling: convenience Data collection year: not reported	1072 nursing students/ not reported Gender: not reported Age: not reported	Knowles, 1975	Items: 20 Dimensions: four (learning motivation, plan and execution, self-monitoring, interpersonal relationship) 5-point Likert scale Scores: from 20 to 100 Language: Chinese
	Cheng et al., [33] Taiwan	1 – To use the IRT with a graded response model to re-examine the SDLI instrument 2 – To establish the SDL ability norms among four nursing education programmes in Taiwan	Design: not reported Sampling: stratified random sampling with probability proportional to size Data collection year: not reported	7879: 667 BSN nursing students, 971 RN-to-BSN students, 5452 ADN students and 789 BS student/15% Female: 93.8% Age: average 18.5 years	Not reported	See above Language: not reported
	Shen et al., [34] China	To test validity and reliability of the SDLI	Design: cross-sectional Sampling: two convenience samples Data collection year: not reported	a.1499 nursing students/99.3% b.30 nursing students (test–retest)/not reported Female: 96.1% Age: range 18–24 years	Not reported	See above Language: not reported

*ADN* Associate Degree in Nursing, *BS* Bachelor of Science, *RN* registered nurses, *RTs* radiology technicians, *RN-to-BSN* Registered Nurses-to-Bachelor in Nursing Science, *SDLI* Self-Directed Learning Instrument, *SDLRS* Self-Directed Learning Readiness Scale, *SDLRSNE* Self-Directed Learning Readiness Scale for Nursing Education, *SRSSDLI* Self-Rating Scale of Self-Directed Learning

All included studies measured SDL abilities among nursing students [3, 11, 24, 29–35], by also including registered nurses [29, 33], and other health-care professionals such as health-care assistants, paediatric nurses, midwives and radiology technicians [28, 29].

The sampling method was mainly based on a convenience sample [24, 28, 29, 31, 34, 35]. However, in two studies all nursing students of the target population were included [11, 30], while in one [33], a stratified random sampling was adopted. In two studies [3, 31] the sampling method adopted was not reported.

Studies have involved from 30 [35] to 7879 [33] participants. Four studies [11, 28, 30, 34] have reported a response rate ranging from 80 to 100%. When reported, participants, were in the majority female, from 75 to 97% [3, 11, 28–31, 33, 34], and their age average was variable, from 18 [33, 34] to 41.2 years [28].

In the above-mentioned studies, four tools based on Knowles’s andragogic theory emerged as reported in Table 3: 1) the Self-Directed Learning Readiness Scale (SDLRS) [11]; 2) the Self-Directed Learning Readiness Scale for Nursing Education (SDLRSNE) [3, 31, 32], 3) the Self-Rating Scale of Self-directed Learning (SRSSDL) [28–30, 35], and 4) the Self-Directed Learning Instrument (SDLI) [24, 33, 34].

All tools are self-report instruments based upon either pencil paper or online format. Only five studies [3, 24, 28–30] have specified the language of the tool, which was Italian [28–30] and Chinese [3, 24].

#### Self-directed learning readiness scale (SDLRS)

The first instrument was the SDLRS tool [11] validated previously by Guglielmino in 1977 among US university students and composed of 57 Likert-type items (from 1 – strongly disagree; to 5 – strongly agree), thus resulting in a score ranging from 57 to 285 [11]: the higher scores indicate higher SDL abilities.

In developing the tool, Guglielmino [22] established eight dimensions of the SDLRS (the items distribution is not available): openness to learning opportunities; self-concept as an effective learner; initiative and independence in learning; informed acceptance of responsibility for one’s own learning; love of learning; creativity; positive orientation to the future; ability to use basic study skills; and problem-solving skills. In his following validation among nursing students, Crook [11] did not report the dimensions of the instrument, whether they were different to the original or not.

#### Self-directed learning readiness scale for nursing education (SDLRSNE)

The second instrument was the Self-Directed Learning Readiness Scale for Nursing Education (SDLRSNE) [3, 31, 32] developed and validated by Fisher in 2001 among nursing students at the University of Sydney, Australia. It was composed of 40 items categorized into three dimensions: self-management (13 items); desire for learning (12 items); and self-control (15 items). Four items were negatively phrased. The response for each item was rated using a five-point Likert scale (from 1 – strongly disagree, to 5 – strongly agree) with a range of total scores from 40 to 200. A total score > 150 was set as a cut-off indicating SDL readiness [32]. Subsequently, the factor structure was re-examined [31] and validated among Japanese nursing students [3].

#### Self-rating scale of self-directed learning (SRSSDL)

The third instrument was the Self-Rating Scale of Self-Directed Learning (SRSSDL). Williamson [35] developed and validated this tool in a sample of nursing students at Thames Valley University, UK. The SRSSDL consists of 60 items categorized into five dimensions: awareness (12 items); learning strategies (12 items); learning activities (12 items); evaluation (12 items); and interpersonal skills (12 items) [35]. The responses for each item are rated using a five-point Likert type scale (5 = always, 1 = never). The score may range from 60 to 300 indicating respondents’ level of SDL abilities: low level (60–140); moderate level (141–220); and high level (221–300). The tool has been subsequently validated in the Italian context by Cadorin and colleagues [28, 29] by involving nurses, health-care assistants, nurses, midwives and radiology technician students and professionals. At the end of the validation process the SRSSDL__ITA_ was established, including 40 items categorized into eight dimensions: awareness (7 items); attitudes (8 items); motivation (6 items); learning strategies (5 items); learning methods (4 items); learning activities (4 items); interpersonal skills (4 items); and constructing knowledge (2 items). The total score may range from 40 to 200 and a higher score indicates a higher level of SDL abilities [29, 30].

#### Self-directed learning instrument (SDLI)

The fourth instrument was the Self-Directed Learning Instrument (SDLI) developed and validated by Cheng and colleagues [24, 33, 34] among Taiwanese nursing students from three representative nursing programmes: Associate Degree in Nursing (ADN), Bachelor of Science in Nursing (BSN) and Registered Nurse (RN) to BSN programme. The instrument consists of 20 items categorized in four dimensions of SDL learning: motivation (6 items); plan and execution (6 items); self-monitoring (4 items); and interpersonal relationships (4 items). The metric is based upon a five-point Likert-type scale (from 1 strongly disagree to 5 strongly agree) and may range from 20 to 100, where higher scores indicate higher levels of SDL abilities.

### Methodology qualities used in the instrument validation processes

As reported in Table 4, five studies estimated only two psychometric properties [3, 11, 29, 31, 32]; the remaining estimated three psychometric properties [24, 28, 30, 33, 35], while only Shen et al. [34] estimated four psychometric properties. All studies assessed internal consistency while measurement error and responsiveness were never estimated.

**Table 4:** Instruments evaluating SDL abilities: psychometric properties^a^ and their methodological quality of evaluation^b^

Tool	Authors, year	Step 2	Internal Consistency	Reliability	Content Validity	Structural Validity	Hypotheses Testing	Criterion Validity	Cross-Cultural Validity
		CTT or IRT	α Cronbach	ICC	Yes	Methods used; main findings	Yes	Tool, *r* Pearson *p* value	Yes
SDLRS	Crook, [11]	–	–	–	–	–	8% variance ++	0.258 *p* < 0.02 ++	–
SDLRSNE	Fisher et al., [32]	–	0.924 ++	–	–	PCA; 36.4% ++	–	–	–
	Fisher and King, [31]	–	0.87 +	–	–	CFA; Self-management RMSEA = 0.039, GFI = 0.960, GFI-AGFI = 0.023, CFI = 0.971, SRMR = 0.039, Desire for learning RMSEA = 0.024, GFI = 0.971, GFI-AGFI =0.020, CFI = 0.993, SRMR = 0.032, Self-control RMSEA = 0.054, GFI = 0.951, GFI-AGFI = 0.028, CFI = 0.930, SRMR =0.031 ++	–	–	–
	Fujino-Oyama et al., [3]	–	0.91 ++	–	–	CFA; SRMR = 0.097, RMSEA = 95%, CI = 0.081 [0.078,0.085], CFI = 0.654, PGFI =0.673 ++	–	–	–
SRSSDL	Williamson, [35]	–	0.71–0.79 +	–	Delphy study +	Known-groups technique; 1st year scores were 160; final year students’ scores were 214 +	–	–	–
	Cadorin et al., [28]	–	0.94 ++	0.73 ++	–	–	–	–	Forward back-translation +
	Cadorin et al., [29]	–	0.929 +	–	–	EFA; 54.304% ++	–	–	–
	Cadorin et al., [30]	–	SRSSDL 0.93 SDLI 0.90 +++	–	–	–	–	SDLI 0.815 p < 0.001 +++	Forward back-translation +
SDLI	Cheng et al., [24]	–	0.916 ++	–	Delphi study ++++	CFA; RMS = 0.04, RMSEA = 0.057, GFI = 0.94, AGFI = 0.92, NFI = 0.93, AIC = 0.76 ++	–	–	–
	Cheng et al., [33]	++++	0.94 ++++	–	–	GRM; discrimination parameter for all items SDLI tool was between 1.41 and 2.99 ++++	–	–	–
	Shen et al., [34]	–	0.91 ++	0.916 ++	–	EFA 53.3%; CFA: RMR = 0.028, RMSEA = 0.057, CFI = 0.930, GFI = 0.929, AGFI = 0.909, PGFI = 0.781, NFI = 0.905 ++	–	SRSSDL 0.876 p < 0.001 ++	–

^a^Measurement error (Box C) and Responsiveness (Box I) were not included in the table given that no studies have estimated these psychometric properties

^b^ + poor; ++ fair; +++ good; ++++ excellent

Legend: *α* Cronbach’s alpha coefficient – Total scale, *AGFI* Adjusted Goodness of Fit Index, *CFA* confirmatory factor analysis, *CFI* comparative fit indices, *CI* confidence interval, *EFA* explorative factor analysis, *GFI* goodness of fit, *GRM* Graded Response Model, *ICC* interclass correlation coefficient, *IRT* Item Response Theory, *NFI* Normed Fit Index, *PCA* principal components analysis, *PGFI* Parsimony Goodness-of-Fit Index, *r* Pearson’s coefficient, *RMR* root mean square residual, *RMS* standardized residual, *RMSEA* root mean square error of approximation, *SDLI* Self-Directed Learning Instrument, *SDLRS* Self-Directed Learning Readiness Scale, *SDLRSNE* Self-Directed Learning Readiness Scale for Nursing Education, *SRMR* standardized root mean square residual, *SRSSDLI* Self-Rating Scale of Self-Directed Learning

Regarding the quality of the above-mentioned estimations as reported in Table 4, only Cheng et al. [24] used excellent methodology quality in all psychometric properties evaluated.

The remaining studies used mostly fair to poor quality of methodologies while one was poor in all boxes evaluated [35]. The major problem affecting the quality of the methodologies and the final scores was regarding the missing items (specifically in boxes A, B, E, F, G, H) and how they were handled by researchers.

### Estimated psychometric properties comparison across instruments

Evaluating the psychometric properties as reported in Table 4, all instruments have reported an internal consistency of α > 0.71; the reliability, when estimated, was from 0.73 [29] to 0.91 [34]. The content validity was performed using a Delphi technique by Williamson [35] and by Cheng et al. [24]. The structural validity explored through different methods has reported good indexes both in the explorative factor analysis (e.g. 54.3% in Cadorin et al. [29]; 53.3% in Shen et al. ([34]) and in the confirmative factor analysis [3, 24, 31, 34]. Criterion validity was also higher among the SDLI and the SRSSDL as measured by Shen et al. (*r* 0.876) [34] and by Cadorin et al. (*r* 0.815) [30].

## Discussion

### Validated instruments evaluating SDL abilities

This is the first psychometric systematic review summarizing the psychometric properties of self-report tools measuring the SDL abilities among nursing students and nurses. According to the findings, only 11 studies developed mainly by interconnected authors (Cadorin, Cheng, Fisher and Williamson) have emerged. However, studies were conducted in different countries, involving mainly nursing students as a convenience sample with a higher response rate, thus suggesting a potential selection bias [36].

The number of participants was mainly below the suggested ratio between the number of items and the respondents (1 item/≥ 10 respondents) [37], which was respected in only four studies [24, 29, 30, 33, 34]. Moreover, participants involved were mostly female, reflecting the gender distribution of the nursing population, which is predicted to change in future years [38], thus suggesting the need to validate future instruments in a more diverse population.

No studies implying international validation in multiple languages have been developed up to now, a gap which needs to be filled given the increased occurrence of emigration among nurses around the world and the encouragement given to educating nursing students internationally [39]. Moreover, participants were mainly younger in those studies involving only nursing students, while the average age was higher (from 34 to 41 years) when nurses and/or other health-care professionals were also involved.

Several data (e.g. when the data collection was performed, age and gender of participants) were missed in the studies, thus potentially affecting the external validity of the findings and suggesting the need to establish a minimum data set when instrument validation is undertaken in this field.

Knowles’s theory framework [10] was the conceptual reference considered in tool development and analysis: this model is the best known in the SDL field and it is considered a ‘*linear model’* given that it describes self-directed learning as a series of steps through which learners make their progress [14]. Different models have also been defined, e.g. ‘interactive models’ where learners’ traits and educational processes interact with each other to develop SDL abilities [40]; and ‘instructional models’ where the educators integrate methods into their programmes aiming at stimulating the students to become responsible, independent and self-directed [41].

The fact that tools have been based on the Knowles theory [10] has great potential for also comparing the validity of instruments across countries and cultures. Moreover, by excluding the study of Crook [11], which was the first based on the Guglielmino’s tool [22], with no further validation having been developed to our best knowledge, the remaining three instruments have been validated by different authors who have refined and re-tested the psychometric estimations and further developed the instruments. Therefore, this field of research seems to be characterized by a cumulative process of research, which may develop more consistent evidence on SDL measurement by reducing dispersion.

Conceptually, while the SDLRSNE [3, 31, 32] evaluates SDL readiness as the degree to which an individual has the characteristics, attitudes, preferences and capabilities required for SDL [3], the SRSSDL [28–30, 35] and the SDLI [24, 33, 34] consider SDL “self-reported” skills or abilities. Therefore, while the first instrument measures individual traits, this second group of tools is aimed at providing an immediate feedback on actual abilities, and may assist in choosing the best strategies as well as in evaluating their effectiveness [28].

A decreased complexity of tools has emerged over the years: from the initial 57 items [11], the SDLRSNE was based on 40 items [32] as well as the SRSSDL in its re-validation [30]; the shorter tool was the SDLI [24] based on only 20 items. A simple tool may increase participant willingness to complete the questionnaire as well as their accuracy [42].

Moreover, different dimensions categorizing the items across instruments despite the common reference of Knowles theory [10] have emerged. ‘Motivation’ and ‘Interpersonal skills/relationships’ are common between the SRSSDL [28–30, 35] and the SDLI tools [24, 33]; also ‘Plan and execution’, which emerged in the SDLI tool, has similarities with ‘Learning strategies’, ‘Methods’ and ‘Activities’, which emerged in the SRSSDL. In fact, according to Cadorin et al. [30] these two instruments have reported higher convergent validity (*r* 0.815, *p* < 0.001). The process of dimension labelling may be affected by the knowledge, values and pedagogical background of researchers involved; thus, strengthening the relationship between reference theory and instrument development and validation processes should be considered in the future with the aim of reducing this variability.

With regard to the metrics used, two methods have emerged: the most common is based on the agree/disagree process, consisting of five degrees; conversely, in validating the SRSSDL, Williamson et al. [35] and Cadorin et al. [28–30] there was also used a five-point Likert scale based on a measure of frequency (from always to never), indicating how often an SDL ability is used. Further reflection on which metrics to adopt in this research field, aimed at avoiding acquiescence bias (participants may agree with statements as presented), social desirability bias (participants may declare that they always use a specific strategy when educators have recommended its use) [43] as well as the tendency to answer in the middle and neutral points range (e.g. 3) [44], should be undertaken.

### Methodology qualities used in the instrument validation processes

Three main points of discussion can be considered from the methodologies quality adopted by researchers in estimating the psychometric properties and from the findings reported regarding these psychometric properties.

Firstly, only a few psychometric properties have been evaluated in each study by authors, from two to four out of the ten considered by the COSMIN checklist [21]. Over time, the same instrument has been re-evaluated in the same psychometric properties in different populations and settings and only in a few cases (e.g. [29, 30, 33, 34]) new psychometric properties have been added. Therefore, to date, all instruments evaluating SDL abilities should be considered incomplete with regard to the process of validation: this may be due to the long process necessary to develop and to validate an instrument and the need to communicate early results to the scientific and professional community by publicizing preliminary findings; on the other hand, this may also be due to ‘salami slicing’ and the pressure on researchers to be productive by publishing articles [45]. In addition, it may be due to the lack of standardization in the field of validation studies, which may be supported only recently by the COSMIN guidelines [21] as well as by other guidelines (e.g., the Standards for Reporting of Diagnostic Accuracy [46]; Guidelines for Reporting Reliability and Agreement Studies which refers to reliability measures [47]). Instead, some psychometric properties such as “cross-cultural validity”, are not requested when the tool is validated in the same culture or country as compared to the original version.

Secondly, the quality of the methodologies used ranged in general from fair to poor in accordance with the *‘worst score counts’* as suggested by the COSMIN system [21]. How researchers have handled the missing items was the major failure, suggesting that more accuracy in the reporting process and findings of validation studies is necessary.

Only one study was performed with excellent methodologies in three dimensions [33] and, in general, studies reported an increased quality of the methodology adopted for validating the same instrument over time: for example, Cadorin et al. [30] have used an increased quality of methodologies as compared with previous studies [28, 29] as did Cheng et al. [24, 33]. This may be due to an increased confidence of researchers in validating tools and an increased thoroughness by journals in reviewing and accepting studies aimed at validating instruments.

Thirdly, psychometric proprieties reported for instruments were in general good. For example, the internal consistency was always included in the range from >0.70 and <0.95 as suggested by Terwee et al. [20]. Structural validity assessed was also good in the indices but the poor quality of the methodologies used threatened comparisons.

### Estimated psychometric properties comparison across instruments

Although to date no gold standard has been established among the SDL instruments by the scientific and professional community [30], the significantly higher correlation between the SDLI [24] and the SRSSDL [28, 29] may suggest that a similar latent concept is measured; given that SDLI psychometric properties have been evaluated as being of higher quality in methodologies adopted [24, 33], the tool may be considered as a gold standard in the nursing field. This endorsement is based upon the psychometric properties of the measures documented in the retrieved studies and does not take into account other attributes of the measures that might be important. e.g., if the measures are more widely reported in the literature, the degree of the conceptual precision of the measure. This tool could also be further developed by using a rigorous multimethod approach with the purpose of overcoming the limitations of a single approach [48].

### Limitations

Several limitations may have affected this review. Regarding the research process adopted, all systematic reviews carry the risk of not identifying all the available studies [49]. Although we have adopted a rigorous method, repeated twice, some studies may have been missed. Moreover, only articles published in the English language were included; instruments developed and published in different languages may also have been missed. In addition, the inter-rater agreement assessed for full text inclusion was good; however, after the first assessment, four studies [2, 25, 26, 50] were excluded because the psychometric characteristics were not reported or they did not include the validation of the instrument.

The studies assessment was performed on the basis of the COSMIN guidelines [20, 21] developed for health status measures and not specifically for nursing education instruments. Moreover, the evaluation was performed by analysing what researchers have documented in the included studies; therefore, in some items, such as cross-cultural validity, a lack of data reported in the studies due to limited space allowed in the scientific journal, may have affected the findings. Not lastly, the ‘worst score counts’ method [27] was applied, emphasizing weaknesses and problems in the quality of methodologies adopted in establishing psychometric properties; therefore, the final picture of the state of the art in the SDL instrument evaluation field may suffer from negativity instead of emphasizing the positive aspects. Furthermore, this review was focused on nursing studies where the population included were typically female; therefore, applying these findings to the male gender [38], should be done with caution.

## Conclusion

This psychometric systematic review summarized the quality of the psychometrics and tools used to evaluate SDL abilities among nursing students and nurses; interestingly, in recent years, tools have been validated in a mixed population by also involving different students and health-care professionals in addition to nurses, reflecting that the self-directed learning measurement is a common concern across disciplines. Only 11 studies have emerged where four tools have been subjected to validation of their psychometric properties. Studies are single-country based, framed on the same Knowles theory [10], and have been developed in recent years, indicating that self-directed learning abilities represent a relatively young field of research.

In general, only some psychometric properties have been validated in the available tools, despite the complexity of their process and the measures required to consider an instrument valid; moreover, with the exception of the SDLI, for which the methodology quality adopted in estimating the psychometric properties was excellent, in other instruments the quality of the methodologies used ranged, in general, from fair to poor. For this reason, on the basis of the findings of our review, SDLI can be recommended for the evaluating of SDL abilities among nursing students and nurses. However, given that also other healthcare students and professionals, e.g., medical students, are expected to possess self-directed learning (SDL) skills to pursue lifelong learning, our findings can also be used in other disciplines as a basis for addressing instrument development and validation.

With the increased relevance of self-directed learning abilities in undergraduate, advanced, and continuing education pathways, rigorous study designs aimed at estimating the psychometric properties of tools in large, inter-professional samples, as well as in different cultural, educational and work settings, are strongly recommended.

## Abbreviations

CDSR: Cochrane Database of Systematic Reviews
CINAHL: Cumulative Index to Nursing and Allied Health
COSMIN: COnsensus-based Standards for the selection of health status Measurement Instruments
CTT Classical: Test Theory
EPOC: Effective Practice and Organization of Care
ERIC: Education Resources Information Centre
HEIs: Higher Educational Institutions
IRT: Item Response Theory
MEDLINE: United States National Library of Medicine
MeSH: Medical Subject Headings
PICOS: Population, Interventions, Comparison, Outcomes and Study Designs
PRISMA: Preferred Reporting Items for Systematic reviews and Meta-Analyses
PROSPERO: International Prospective Register of Systematic Reviews – Centre for Review and Dissemination University of York
SCOPUS: Bibliographic database by Elsevier
SDL: Self-directed learning
SDLI: Self-Directed Learning Instrument
SDLRS: Self-Directed Learning Readiness Scale
SDLRSNE: Self-Directed Learning Readiness Scale for Nursing Education
SRSSDL: Self-Rating Scale of Self-directed Learning
US: United States
